# *Toxoplasma gondii* Infection and Chronic Liver Diseases: Evidence of an Association

**DOI:** 10.3390/tropicalmed1010007

**Published:** 2016-11-01

**Authors:** Nagwa Mostafa El-Sayed, Manar Ezzelarab Ramadan, Mohamed Ezzelarab Ramadan

**Affiliations:** 1 Medical Parasitology Department, Research Institute of Ophthalmology, Giza 12556, Egypt; 2 Parasitology Department, National Hepatology and Tropical Medicine Research Institute, Cairo 11796, Egypt; manarezz98@yahoo.com; 3 Department of Hepatology and Gastroenterology, Ahmed Maher Teaching Hospital, Cairo 11638, Egypt; ezzm3@yahoo.com

**Keywords:** toxoplasmosis, chronic liver diseases, hepatitis B virus (HBV), hepatitis C virus (HCV), real-time PCR

## Abstract

Toxoplasmosis may present as a severe disease among some Egyptian patients with chronic liver disease (CLD) due to their impaired immune system, changing the course of the disease. The classical diagnosis of toxoplasmosis by serological tests is inadequate for such patients. This study was performed to highlight the role of real-time quantitative PCR (qrtPCR) test in the accurate diagnosis of toxoplasmosis among Egyptian patients with CLD. Seventy patients with CLD and 50 healthy controls were enrolled in this study. All were subjected to full clinical examinations, abdominal ultrasonography, and biochemical analysis of liver enzymes and they were investigated for markers of hepatitis B virus (HBV) and hepatitis C virus (HCV). In addition, *Toxoplasma gondii (T. gondii)* parasitemia was determined using qrtPCR. The results showed that *T. gondii* parasitemia was positive in 30% of CLD patients with highly statistically significant (*p* < 0.001) compared with the control group (6%). Co-infection in both *T. gondii*/HBV and *T. gondii*/HCV was 33.3% and 31.4%, respectively, with a highly significant association between *T. gondii* parasitemia and HCV viral load. Moreover, the results showed a significant increase of liver enzymes in the serum of patients positive for *T. gondii* compared with negative patients. An association between *T. gondii* infection and CLD was observed, and further studies will be needed to define the mechanism of this association.

## 1. Introduction

*Toxoplasma gondii*, an obligate intracellular protozoan, is the most frequent protozoan causing opportunistic infections in immunocompromised patients [1]. In immunocompetent individuals, the infection is self-limiting because an efficient immune control limits the dissemination of the rapidly multiplying tachyzoite stage [2]. However, the parasite still remains viable in the tissue cysts throughout the whole life of the host. During this stage, cellular immunity mediated by T cells and macrophages, and the activity of type 1 cytokines (IL-12 and IFNγ), plays a crucial role in controlling the tissue cysts and the development of chronic *T. gondii* infection [3,4]. Chronically infected individuals who possess defects in cell-mediated immunity are at risk for reactivation of the infection and its dissemination, causing serious complications with the occurrence of high morbidity and mortality rates among these patients [5].

Chronic liver disease (CLD) is a disease of the liver resulting from an inflammatory, infiltrative, immunologic, mechanical, or metabolic injury to the liver that has persisted for six months or more without complete resolution. Patients with CLD are often highly susceptible to opportunistic parasitic infections due to a depressed immune system [6]. Therefore, opportunistic toxoplasmosis can cause more frequent and severe effects in these patients and can change the course of the disease [7]. According to previous clinical and epidemiological studies, some reports highlighted a potential association between *T. gondii* infection and CLD [8,9]. However, these studies are based on serological tests that are inefficient and inadequate for detecting active infection in immunodepressed subjects [10,11]. The use of molecular diagnosis is particularly appropriate for such patients, since it does not depend on the immunological status of the host. *B1* gene PCR protocol appears to be the most sensitive and specific method for the diagnosis of toxoplasmosis [12]. In this study, a real-time quantitative PCR (qrtPCR) test for accurate diagnosis of toxoplasmosis was performed to highlight the possible association between *T. gondii* infection and chronic liver diseases among Egyptian patients.

## 2. Subjects and Methods

### 2.1. Study Type

Case control study.

### 2.2. Subjects

Seventy patients with CLD (37 males and 33 females, with an age range from 19 to 66 years) were selected from the attenders in the Hepatology and Gastroenterology department at Ahmed Maher Teaching Hospital, Cairo, Egypt, during the period from May 2015 to January 2016. Regarding the etiology of CLD, patients were classified as having chronic hepatitis C virus (HCV) (*n* = 54 patients) or chronic hepatitis B virus (HBV) (*n* = 3 patients), or other causes including biliary atresia, autoimmune hepatitis, hydatid disease, and drug-induced hepatitis (*n* = 13 patients). Moreover, 50 healthy subjects (28 male and 22 females), with the same age range, were enrolled in this study. They were selected from a group of healthcare workers, and full histories, clinical examinations and all investigations were done, to detect the possibility of CLD. Informed consent was obtained from each participant, and the study was approved by the Committee of Research, Publications and Ethics of Ahmed Maher Teaching Hospital, Cairo, Egypt.

Blood samples were collected from all participants, and sera were obtained via centrifugation, frozen down, and kept stored at −20 °C until analyzed.

### 2.3. Investigations

#### 2.3.1. Abdominal Ultrasonography

Abdominal ultrasonography was done to assess texture and size of liver, size of spleen, portal vein diameter, and the presence of ascites. These ultrasound variables/parameters were adopted from the published literature [13,14].

#### 2.3.2. Biochemical Analysis

Sera samples were tested for liver enzymes using kits for aspartate aminotransferase (AST), alanine aminotransferase (ALT) (Randox Laboratories Ltd., Antrim, UK), and alkaline phosphatase (ALP) (BioMerieux, Lyon, France) according to the manufacturer’s instructions.

#### 2.3.3. Detection of Hepatitis Virus Markers

Sera samples were also screened for hepatitis virus markers by enzyme-linked immunosorbent assays (ELISAs) using a hepatitis B surface antigen (HBsAg) assay, (Monolisa HBsAg ULTRA, Bio-Rad Laboratories, Inc., Hercules, CA, USA) for the detection of HBV, and anti-hepatitis C virus antibodies (Ortho HCV version 3.0; Bio-Rad Laboratories Inc., Hercules, CA, USA) for the detection of HCV.

#### 2.3.4. Determination of Viral Load

Determination of HCV and HBV viral load was done by the Amplicor HCV monitor test, version 2.0 (Roche Molecular Systems, Pleasanton, CA, USA), and by the Cobas Amplicore HBV monitor test (Roche Diagnostic Systems, Inc., Somerville, NJ, USA), respectively, according to the manufacturer’s instructions. The viral load was classified into mild viremia (<1,000,000 copies/mL), moderate viremia (1,000,000–5,000,000 copies/mL), and severe viremia (>5,000,000 copies /mL) according to Razzaq and Malik [15].

#### 2.3.5. Detection of the *T. gondii B1* Gene by Real-Time Quantitative PCR (qrtPCR)

*T. gondii* DNA was extracted from serum specimens using QIAamp DNA Blood Mini Kit (Qiagen, Hilden, Germany) following the manufacturer’s instructions. The extracted DNA concentration was confirmed with a NanoDrop 2000c spectrophotometer. Readings were taken at wavelengths of 260 and 280 nm. Concentration of DNA samples was measured, yielding 50 ug mL^−1^ × A260. Amplification was performed using two primers with the following sequences: 5′-AACGGGCGAGTAGCACCTGAGGAGA-3 and 5′-TGGGTCTACGTCGATGGCATGACAAC-3′, which specifically amplified a 115 bp sequence of the *T. gondii* *B1* gene [12]. The used master mix was SuperReal PreMix Plus (SYBR Green) TIANGENE Biotech Co., Ltd. (Beijing, China). Two microliters of DNA extract were added in a 20 µL reaction volume. In the ABI7900 fast real time machine (Applied Biosystem, Foster City, CA, USA), the assays were designed to detect the *T. gondii* in each sample using its specific primers in addition to the glyceraldehyde phosphate dehydrogenase gene (GAPDH) as an internal positive control with the following sequences; 5′-TGA TGA CAT CAA GAA GGT GGT GAA G-3′ and 5′-TCC TTG GAG GCC ATG TGG GCC AT-3′. The program conditions were 95 °C, 15 min for initial denaturation followed by 35 cycles of 95 °C, 30 s; 54 °C, 1 min.; 72 °C, 30 s. Positive and negative controls were used for each run. The positive control was *T. gondii* DNA extracted from RH strain tachyzoites, kindly provided by the Department of Zoonotic Diseases, Veterinary Research Division, National Research Center, Giza, Egypt, while the negative control was a blank containing all PCR reagents without DNA. The cycle threshold value, indicative of the quantity of the target gene at which the fluorescence exceeds a preset threshold, was determined. This threshold was defined as 20 times the standard deviation of the baseline fluorescent signal. After reaching the threshold, the sample was considered positive.

### 2.4. Statistical Analysis

Data analysis was performed using SPSS 16.0 (SPSS Inc., Chicago, IL, USA). The findings were expressed as percentage and mean ± standard deviation. The data were evaluated via Pearson’s chi-square test and the Student’s *t*-test (*t*). Values were considered statistically significant when *p* < 0.05.

## 3. Results

### 3.1. Association between T. gondii Positivity and the Etiology of CLD

In the present study, the etiology of CLD was shown to be chronic HBV in 3 (4.3%) cases, chronic HCV in 54 (77.1%) cases, and other causes in 13 (18.6%) cases, which included biliary atresia, autoimmune hepatitis, hydatid disease, and drug-induced hepatitis. However, all selected controls were proven to be free of any liver diseases. The clinical presentations of the studied patients are recorded in Table 1 and include focal lesions, splenomegaly, ascites, enlarged or shrunken liver, and dilated portal vein. *T. gondii* parasitemia was positive in 30% of CLD patients and was highly statistically significant compared with the control group (6%) (Table 2). The risk of co-infection i.e. *T. gondii*/HBV and *T. gondii*/HCV was 33.3% and 31.4%, respectively; however, this association did not reach a significant value (Table 3).

### 3.2. Association between T. gondii Parasitemia and Viral Load

Regarding HCV load, *T. gondii* parasitemia was positive in 13.8%, 50.0%, and 66.7% of patients with mild, moderate and severe viremia, respectively. There was a highly significant association between *T. gondii* positivity and a higher viral load. *T. gondii* parasitemia was positive in 33.3% of HBV patients with moderate viremia, and there were no cases detected with respect to mild and high viral load (Table 4).

### 3.3. Association between T. gondii Positivity and Liver Enzymes

The results showed a significant increase of liver enzymes: AST, ALT, and ALP in the sera of positive toxoplasmosis patients compared with negative patients. In controls, liver enzyme levels were within a normal range in *Toxoplasma* negative and were increased in *Toxoplasma* positive (Table 5).

## 4. Discussion

Patients with chronic liver disease are liable to a wide spectrum of bacterial, viral, and parasitic infections [16]. Viral hepatitis represents one of the most common etiologies of liver disease in Egypt. In the present study, HCV was found to be a major cause among Egyptian patients with CLD (77.1%); for HBV, 4.3%. This is apparently due to past parenteral antischistosomal therapy [17].

Most forms of CLD are accompanied by the depression of both humoral and cell-mediated immunity, with a marked inability of handling invading pathogens. During the parasitemia stage of *T. gondii* infection, the liver is one of the most important organs involved and affected [18]. The mechanisms of liver damage and the histological changes induced by *T. gondii* infection could be due to a direct proliferative effect of the parasite on the tissues, leading to cell death and tissue damage, or could be related to the indirect effect of infection due to the excessive immunological response to the parasite [19].

Experimental studies observed the presence of tachyzoites and tissue cysts inside the hepatocytes and within the sinusoidal liver capillaries. When such a parasite invades the hepatocyte, it can lead to disturbances in its metabolic activity, shape distortion, and damage in its DNA [20,21]. It is known that the parasite locates the host liver and causes pathological changes that progress to hepatomegaly, granuloma, hepatitis, and necrosis [2]. Additionally, there is a significant relationship between the number of hepatic stellate cells (HSCs) and *T. gondii* antigens, which may represent an active role of HSCs in liver pathology and the pathobiology of *T. gondii*-related hepatitis [22].

The serological diagnosis of toxoplasmosis in immunodeficient patients has important limitations, as the underlying immunosuppression alters antibody production and its kinetics. Therefore, the demonstration of parasites in blood, body fluids, and tissues is the mainstay of diagnosis, providing definitive proof of the disease [23]. In blood, the parasite is found during the phase of parasitemia early in the acute phase of infection and in reactivating disseminated cases [24]. In the present study, the qrtPCR assay was used for detection of the *Toxoplasma B1* gene in blood samples. Chabbert et al. [25] validated the choice of *B1* gene-based PCR assays, as these assays clearly appeared to be more sensitive than assays targeting the single-copy P30 gene. The sensitivity of PCR for purified *T. gondii* DNA has been found to be very high because the *B1* gene contains 30–35 copies of repetitive sequences in every tachyzoite [12]. Similarly, Lin et al. [26] stated that qPCR could detect the *Toxoplasma B1* gene at a concentration as low as 0.05 tachyzoite in a 50 µL reaction volume. Moreover, the use of real-time PCR avoids the risk of false positivity due to DNA carryover [27].

The results of this study revealed a higher *T. gondii* parasitemia in CLD patients (30%) compared with the control subjects (6%), with a statistically significant difference. This difference may be explained by the occurrence of humoral and cell-mediated immunity defects in chronically infected patients with a consequent reactivation of latent infection, resulting in the release of *T. gondii* tachyzoites or DNA from the dominant cysts in these tissues and causing *T. gondii* parasitemia [28]. On the other hand, *T. gondii* DNA was detected in 6% of controls without any manifestations of toxoplasmosis, in agreement with El-Sayed et al. [29] who found that 6% of healthy voluntary blood donors had *T. gondii* parasitemia. It is possible that *Toxoplasma* tissue cysts can occasionally rupture without any clinical symptoms in immunocompetent hosts with the release of bradyzoites, a form of *Toxoplasma* parasite with low levels of metabolic activity, into the blood [30].

In the current study, the risk of co-infection i.e. *T. gondii*/HBV and *T. gondii*/HCV was 33.3% and 31.4%, respectively. Moreover, there was a highly significant association between *T. gondii* parasitemia and HCV viral load; however, negative results for toxoplasmosis were associated with lower viral load. These results may be due to the depletion of cell-mediated immunity during chronic infection with HCV and HBV that results in reactivation of the latent *T. gondii* infection [31]. In agreement with the results of this study, El-Nahas et al. [9] ascertained this association in which anti-*T. gondii* IgG antibodies were detected in 92.6% of late-stage cirrhotic patients and in 76.9% of the chronic HCV non-cirrhotic patients (early-stage). Similarly, in an Egyptian study, Ghanam et al. [32] found a 65.5% seroprevalence of *T. gondii* antibodies in patients with acute and chronic hepatic diseases. On the other hand, Alvarado-Esquivel et al. [7] did not find any association between seropositivity to *T. gondii* and cirrhosis. The use of different laboratory methods and matching procedures, the difference in the ages of participants, and the proportions of controls and patients in the different studies might explain the differences in the results.

Serum AST and ALT activities are excellent markers of hepatocellular injury. Normally, these enzymes are present in the liver and other tissues where they function in energy metabolism involving the transamination of amino acids. However, in cases of cellular damage, AST and ALT could leak out into the general circulation leading to elevated activity [33]. Hepatic injury is a well-established complication of toxoplasmosis, as this infection can cause round cell infiltration in the portal areas, cholestasis, swollen endothelial cells, and focal necrosis of liver cells [34]. Moreover, protein fractions of AST and ALT varied according to the intensity of inflammation induced by *Toxoplasma* infection. In this study, there was a significant elevation in the level of liver enzymes in *Toxoplasma* positive patients compared with *Toxoplasma* negative patients in agreement with the findings of Limdi and Hyde [35] and Mahmood and Dawood [36]. Moreover, serum ALP activity was significantly higher in *Toxoplasma* positive patients than that in *Toxoplasma* negative patients. This finding could be explained by the presence of *T. gondii* in the bile duct cells, since hepatic ALP is reported to be present on the canalicular and luminal domain on the bile duct epithelium [35].

In conclusion, there was an association between *T. gondii* infection and chronic liver diseases, and further studies will be needed to define the mechanism of this association.

## Figures and Tables

**Table 1:** Clinical data of the studied chronic liver diseases (CLD) patients. HBV: hepatitis B virus; HCV: hepatitis C virus.

Variable		CLD (70)			Total (70)
		HBV (3)	HCV (54)	Other Causes (13)	
Focal lesions	Negative	3	52	13	68
	Positive	-	2	-	2
Splenomegaly	No	3	11	10	24
	Mild	-	36	2	38
	Moderate	-	7	1	8
Ascites	No	3	38	13	54
	Mild	-	15	-	15
	Moderate	-	1	-	1
Liver	Average	0	2	0	2
	Enlarged	3	15	5	23
	Shrunken	-	37	8	45
Portal vein	Normal	3	51	12	66
	Dilated	-	3	1	4

**Table 2:** Percentage of *T. gondii* parasitemia in CLD patients and the controls.

PCR	CLD Patients		Controls		Chi Square	*p*-Value
	No	%	No	%		
Positive	21	30%	3	6%	10.5	0.001 *
Negative	49	70%	47	94%		
Total	70	100%	50	100%		

* Significant association between *T. gondii* parasitemia and CLD.

**Table 3:** Co-infection between *T. gondii* positivity and viral hepatitis (HBV, HCV).

CLD Etiology	No	*Toxoplasma* Parasitemia				*p*-Value
		Positive		Negative		
		No	%	No	%	
Chronic hepatitis B virus (HBV)	3	1	33.3%	2	66.6%	0.89753 *
Chronic hepatitis C virus (HCV)	54	17	31.4%	37	68.5%	0.61925 *
Other causes	13	3	23.1%	10	76.9%	0.54608 *
Total	70	21	30%	49	70%	

* No significant association between *T. gondii* parasitemia and HBV and HCV (*p* > 0.05).

**Table 4:** Association between *T. gondii* positivity and viral load.

Degree of Viremia		No	*T. gondii* Positivity (18) No (%)	*p*-Value
HCV (54)	Mild	29	4 (13.8%)	0.009 *
	Moderate	22	11 (50.0%)	
	High	3	2 (66.7%)	
HBV (3)	Mild	0	0	NA **
	Moderate	3	1 (33.3%)	
	High	0	0	

* Significant, *p* < 0.05.; ** Not Applicable.

**Table 5:** Effect of *T. gondii* infection on the levels of liver enzymes in CLD patients and controls. AST: aspartate aminotransferase; ALT: alanine aminotransferase; ALP: alkaline phosphatase (ALP).

Liver Enzymes	*CLD* Patients		Controls	
	*Toxoplasma* + ve (21)	*Toxoplasma* − ve (49)	*Toxoplasma* + ve (3)	*Toxoplasma* − ve (47)
	M ± SD	M ± SD	M ± SD	M ± SD
ALT (IU/L)	62.6 ± 11.28 *	42 ± 8.42	52.3 ± 4.42 *	23.2 ± 5.61
AST (IU/L)	78 ± 10.26 *	53.49 ± 10.2	67.9 ± 4.36 *	37.4 ± 1.15
ALP (IU/L)	81.2 ± 0.7 *	68.1 ± 1.8	88.7 ± 2.12 *	74.1 ± 5.19

* Significant increase of the liver enzymes in the serum of positive toxoplasmosis in both patients and controls compared with negative toxoplasmosis subjects (*p* < 0.0001).
